# Aerial View of the Association Between m6A-Related LncRNAs and Clinicopathological Characteristics of Pancreatic Cancer

**DOI:** 10.3389/fonc.2021.812785

**Published:** 2022-01-03

**Authors:** Bowen Huang, Jianzhou Liu, Jun Lu, Wenyan Gao, Li Zhou, Feng Tian, Yizhi Wang, Mingjie Luo, Dong Liu, Congyong Xie, Ziyu Xun, Chengxi Liu, Yu Wang, Haibo Ma, Junchao Guo

**Affiliations:** ^1^ Department of General Surgery, State Key Laboratory of Complex Severe and Rare Diseases, Peking Union Medical College Hospital, Chinese Academy of Medical Sciences and Peking Union Medical College, Beijing, China; ^2^ State Key Laboratory of Molecular Oncology, National Cancer Center/National Clinical Research Center for Cancer/Cancer Hospital, Chinese Academy of Medical Sciences and Peking Union Medical College, Beijing, China; ^3^ Department of Mathematics, Jinan University, Guangzhou, China; ^4^ Department of Liver Surgery, State Key Laboratory of Complex Severe and Rare Diseases, Peking Union Medical College Hospital, Chinese Academy of Medical Science and Peking Union Medical College, Beijing, China

**Keywords:** pancreatic cancer, m6A, lncRNA, prognostic model, clinicopathological characteristics

## Abstract

Pancreatic cancer is a highly malignant tumor with a poor survival prognosis. We attempted to establish a robust prognostic model to elucidate the clinicopathological association between lncRNA, which may lead to poor prognosis by influencing m6A modification, and pancreatic cancer. We investigated the lncRNAs expression level and the prognostic value in 440 PDAC patients and 171 normal tissues from GTEx, TCGA, and ICGC databases. The bioinformatic analysis and statistical analysis were used to illustrate the relationship. We implemented Pearson correlation analysis to explore the m6A-related lncRNAs, univariate Cox regression and Kaplan-Meier methods were performed to identify the seven prognostic lncRNAs signatures. We inputted them in the LASSO Cox regression to establish a prognostic model in the TCGA database, verified in the ICGC database. The AUC of the ROC curve of the training set is 0.887, while the validation set is 0.711. Each patient has calculated a risk score and divided it into low-risk and high-risk subgroups by the median value. Moreover, the model showed a robust prognostic ability in the stratification analysis of different risk subgroups, pathological grades, and recurrence events. We established a ceRNA network between lncRNAs and m6A regulators. Enrichment analysis indicated that malignancy-associated biological function and signaling pathways were enriched in the high-risk subgroup and m6A-related lncRNAs target mRNA. We have even identified small molecule drugs, such as Thapsigargin, Mepacrine, and Ellipticine, that may affect pancreatic cancer progression. We found that seven lncRNAs were highly expressed in tumor patients in the GTEx-TCGA database, and LncRNA CASC19/UCA1/LINC01094/LINC02323 were confirmed in both pancreatic cell lines and FISH relative quantity. We provided a comprehensive aerial view between m6A-related lncRNAs and pancreatic cancer’s clinicopathological characteristics, and performed experiments to verify the robustness of the prognostic model.

## Introduction

Of all the primary human cancers, pancreatic cancer has the worst prognosis. In the United States, approximately 57,600 people are diagnosed with pancreatic cancer each year, and 47,000 people die from this disease, ranking as the third leading cause of cancer death after lung cancer and colorectal cancer (1), with a 5-year survival rate of 6% (2). After decades of fighting pancreatic cancer, surgical resection remains the only possible cure. Unfortunately, due to the late onset of clinical symptoms in pancreatic cancer patients, only 15%-20% of patients have the opportunity to undergo pancreatic resection, and the postoperative 5-year survival rate is only 18% (3). Patients who can’t receive surgical treatment also can’t benefit from chemical drugs, possibly because most patients with chemotherapy already have locally advanced or metastatic nidus. There is also the difficulty in diagnosing pancreatic cancer, often delayed due to the early stage’s lack of symptoms. Therefore, it is imperative to have sensitive and accurate molecular markers in the early diagnosis, prognosis judgment, and treatment strategy selection of pancreatic cancer (4).

Most studies have suggested that N6-methyladenosine(m6A) methylation, one of the most common RNA modifications, can affect the complexity of cancer progression by regulating biological functions related to cancer. m6A modification of noncoding RNAs regulates cleavage, transport, stability, and degradation (5). The m6A regulators can be divided into three types: writers (methyltransferases), readers (signal transducers), and erasers (demethylases) (6). Recent research has demonstrated that m6A modification could regulate tumorigenesis and progression in pancreatic cancer. For instance, The writer METTL3 promotes pancreatic cancer cell proliferation, invasion, chemoresistance, and radioresistance (7, 8). The upregulation of reader HNRNPC was associated with rs7495G, which confer a higher risk of PDAC through a miRNA-mediated manner (9). The eraser ALKBH5 prevents pancreatic cancer progression by posttranscriptional activation of PER1 through m6A abolishment, decreasing WIF-1 RNA methylation and mediating Wnt signaling (10, 11).

It’s well known that long non-coding RNAs’ (lncRNAs) abnormal expression is closely connected with the degree of tumor malignancy. Very little research found that m6A modification can affect pancreatic cancer progression by interfering with the expression of lncRNAs so far (12, 13). Our team hoped to identify the prognostic significance of m6A-related lncRNAs by bioinformatics and statistical analysis of data from patients with PDAC based on Genotype-Tissue Expression(GTEx), The Cancer Genome Atlas(TCGA), and International Cancer Genome Consortium(ICGC) databases. Furthermore, we constructed an m6A-related lncRNAs prognostic model to predict the overall survival of PDAC patients. Meanwhile, the stratified analysis was carried out with PDAC patients in different risk and clinicopathological subgroups, categorized based on the lncRNAs prognostic model. Furthermore, we built a competing endogenous RNA(ceRNA) network to search the target miRNAs and m6A regulators of these m6A-related prognostic lncRNAs. Besides, we identified small molecule drugs that may interfere with pancreatic cancer progression by targeting mRNA expression levels. Ultimately, we also explored whether critical lncRNAs were differentially expressed between tumor and normal samples in the GTEx-TCGA database, cell lines, and tissue microarray. In a word, we have drawn a bird-eye view of the relationship between m6A-related lncRNAs and clinicopathological characteristics of pancreatic cancer.

## Materials and Methods

### Databases and m6A Regulatory Genes

We merge the GTEx and TCGA databases as the training set, Fragments Per Kilobase of transcript per Million mapped reads (FPKM) normalized RNA-seq and the corresponding clinicopathological data were acquired from the University of California, Santa Cruz (UCSC) website (https://xenabrowser.net/datapages/). To obtain an ICGC validation set, we downloaded standardized RNA-seq data and related clinicopathological profiles from the ICGC website (https://daco.icgc.org/). We got a GTEx-TCGA training set involving 178 patients and 171 normal samples and an ICGC-CA validation dataset involving 262 patients. Patients with missing critical lncRNAs expression profiles, survival data, and clinicopathological features were excluded in clinical data analysis.

To include m6A regulatory genes that have been experimentally confirmed as much as possible, we searched PubMed for all literature associated with m6A modification. Several reviews comprehensively summarized that all the m6A regulatory genes were adopted (6, 14–17). Finally, twenty-six genes were included in subsequent studies, including ten writers, fourteen readers, and two erasers (**Table 1**).

**Table 1:** The list of the 26 m6A-related methylation regulative factors from publications.

m6A Type	Regulator	Gene Synonyms	Ensembl ID
Writer	METTL3	M6A, MT-A70, Spo8	ENSG00000165819
	METTL14	KIAA1627	ENSG00000145388
	METTL16	METT10D, MGC3329	ENSG00000127804
	ZCCHC4	FLJ23024, HSPC052, ZGRF4	ENSG00000168228
	WTAP	KIAA0105, MGC3925, Mum2	ENSG00000146457
	VIRMA	DKFZP434I116, KIAA1429, fSAP121	ENSG00000164944
	RBM15	OTT, OTT1	ENSG00000162775
	RBM15B	HUMAGCGB, OTT3	ENSG00000259956
	ZC3H13	DKFZp434D1812, KIAA0853, Xio	ENSG00000123200
	CBLL1	FLJ23109, HAKAI, RNF188	ENSG00000105879
Reader	YTHDF1	C20orf21, FLJ20391	ENSG00000149658
	YTHDF2	CAHL, HGRG8, NY-REN-2	ENSG00000198492
	YTHDF3	FLJ31657	ENSG00000185728
	YTHDC1	KIAA1966, YT521, YT521-B	ENSG00000083896
	YTHDC2	DKFZp564A186, FLJ10053, FLJ2194	ENSG00000047188
	IGF2BP1	IMP-1	ENSG00000159217
	IGF2BP2	IMP-2	ENSG00000073792
	IGF2BP3	CT98, IMP-3, IMP3	ENSG00000136231
	HNRNPC	HNRPC	ENSG00000092199
	RBMX	HNRNPG, RNMX, hnRNP-G	ENSG00000147274
	HNRNPA2B1	HNRPA2B1	ENSG00000122566
	EIF3A	EIF3, EIF3S10, KIAA0139, TIF32	ENSG00000107581
	FMR1	FMRP, FRAXA, MGC87458, POF, POF1	ENSG00000102081
	PRRC2A	BAT2, D6S51E, G2	ENSG00000204469
Eraser	FTO	ALKBH9, KIAA1752, MGC5149	ENSG00000140718
	ALKBH5	FLJ20308, OFOXD1	ENSG00000091542

### Annotation of LncRNAs

The lncRNA annotation file of Genome Reference Consortium Human Build 38 (GRCh38) release 102 was acquired from the Ensembl website (http://asia.ensembl.org/index.html) for annotation of the lncRNAs in the GTEx-TCGA and ICGC databases. By recognizing the genes’ Ensemble IDs, We could identify the lncRNAs in the GTEx-TCGA and the ICGC databases.

### Cell Culture

Six human PDAC cell lines, AsPC-1, BxPC-3, Capan-1, CFPAC-1, MIA PaCa-2, SW1990, and one normal pancreatic duct epithelium cell line, HPNE, were obtained from the American Type Culture Collection (ATCC) (Manassas, VA, USA). Capan-1 and CFPAC-1 were cultured in Iscove’s Modified Dulbecco Medium (IMDM) (HyClone, Logan, UT, USA), SW1990 was cultured in Roswell Park Memorial Institute (RPMI) 1640 Medium (HyClone, Logan, UT, USA), and the rest of cell lines were cultured in Dulbecco’s Modified Eagle Medium (DMEM) (HyClone, Logan, UT, USA). The Capan-1 was supplemented with 20% fetal bovine serum (FBS) (Gibco, CA, USA), and the rest of the cell lines were supplemented with 10% FBS. All of them were at 37°C in a 5% CO2 cell culture incubator.

### RNA Reverse Transcription and Quantitative Real-Time Polymerase Chain Reaction(qRT-PCR)

Cells were plated in 6-well plates at 5 × 10^5^ cells/well and total RNA was extracted from the cell lines with TRIzol™ Reagent (15596026; Life Technologies Corporation, Carlsbad, CA, USA) and total RNA was reverse transcribed using a riboSCRIPT™ mRNA/lncRNA qRT-PCR Kit (Ribobio, Guangzhou, China) to synthesize cDNA. The obtained cDNAs were quantified by RT-PCR using a Veriti^®^ 96-Well Thermal Cycler (4375786; Applied Biosystems, Foster City, CA, USA). All steps were according to the manufacturer’s instructions. Glyceraldehyde-3-phosphate dehydrogenase (GAPDH) was used as endogenous controls for lncRNAs. All samples were normalized to internal controls, and the fold changes were calculated using a relative quantification method (2^−ΔΔCt^). qRT-PCR reactions were performed in triplicate. The primer sequences were listed in **Supplementary Table 1**.

### Tissue Microarray Construction and Fluorescence *In Situ* Hybridization (FISH)

The tissue microarray (Outdo, Shanghai, China) was established to detect the expression level of lncRNAs in PDAC tissues and paracancer tissues by processing formalin-fixed and paraffin-embedded blocks. FISH staining was performed using primers (Tsingke, Beijing, China) at 500nM. Two experienced pathologists independently performed the staining evaluation. FISH relative quantity was applied to evaluate the expression level of LncRNAs according to the staining intensity and proportion score among positive-stained cells using a scale of quartering. Tissues without FISH staining were not included in the statistics.

### Bioinformatic Analyses

The Pearson correlation analysis was applied to mining m6A-related lncRNAs. We defined the | Pearson R| > 0.6 and p < 0.001 as the criteria to extract m6A-related lncRNAs. Then univariate Cox regression and Kaplan-Meier (K-M) analyses were implemented to filtrate the prognostic m6A-related lncRNAs in the two databases. We use the Venn diagram to extract the pivotal lncRNAs that can satisfy the screening of two databases and two methods. The correlational relationships among the m6A related-lncRNAs in PDAC were analyzed based on Spearman’s correction coefficient calculation. Moreover, using the R package “glmnet” to conduct Least Absolute Shrinkage and Selection Operator (LASSO) Cox regression (18), we could establish an m6A-related lncRNA prognostic model for the pancreatic cancer patients. The risk score calculating equation is:


$$Riskscore=\sum_{k=1}^{n}Coef_{k}\times x_{k}$$

Which *Coef_k_* means the coefficients, *x_k_* is the FPKM value of each prognostic lncRNAs. Risk scores were calculated for all PDAC patients involved in our project. Using the GTEx-TCGA cohort, Differentially Expressed Genes (DEG) in the high-risk subgroup PDAC patients in contrast to the low-risk subgroup were identified based on the standards of | log2(fold change)| > 0.5 and p < 0.05 using the R package “limma” (19). The DEG of the tumor and normal tissues in different subgroups according to the criterion of | log2(fold change)| > 2 and p < 0.01 in GETx-TCGA databases were shown used by the R package “limma” too. The R packages “clusterProfiler” and “org.Hs.eg.db” were library to Gene Ontology (GO) and Kyoto Encyclopedia of Genes and Genomes (KEGG) Pathway analyses. Python language was employed to predict the target miRNAs of the m6A-related lncRNAs in the miRcode (http://www.mircode.org/) and Starbase (http://starbase.sysu.edu.cn/) databases. What’s more, we searched for the target mRNAs of these miRNAs in miRDB (http://mirdb.org/), miRTarBase (http://mirtarbase.cuhk.edu.cn/php/index.php), and Starbase databases. The ceRNA network was plotted using the software of “Cytoscape” (20). Lastly, we used the Connectivity Map (CMAP) database (https://portals.broadinstitute.org/cmap/) to find out the small molecule drugs that might be related to PDAC following the genes that were distinctly different between tumor and normal samples mentioned above (21).

### Statistical Analyses

The K-M curves and the log-rank test were utilized to compare all genes’ overall survival and extract the m6a-related prognostic lncRNAs. The prognostic ability of the model for 1/3/5-year overall survival was evaluated by receiver operating characteristic (ROC) curves and the area under the curve (AUC) values (22). The Kruskal-Wallis test was used to compare the risk scores between different subgroups in the TCGA database based on the following clinicopathological features: age, gender, smoking, drinking, diabetes, pancreatitis, grade, stage, TNM classification, location, recurrence, outcome, new tumor, multi-malignancies. The student’s t-test was used to compare the seven lncRNAs expression among six PDAC cell lines and one normal pancreatic duct epithelium cell line. Univariate and multivariate Cox regression analyses were employed to assess the independent prognostic value of the m6A-related lncRNAs prognostic model regarding overall survival in two cohorts. The Mann-Whitney test was used to evaluate the lncRNAs relative quantity between PDAC tissue and paracancer normal tissues. All statistical data and figures were analyzed by R (version 4.0.3) and GraphPad Prism 8.0 to ensure aesthetics and editability. All statistical results with a p-value <0.05 were considered significant.

## Results

### Identification of m6A-Related LncRNAs in PDAC Patients

**Figure 1:**
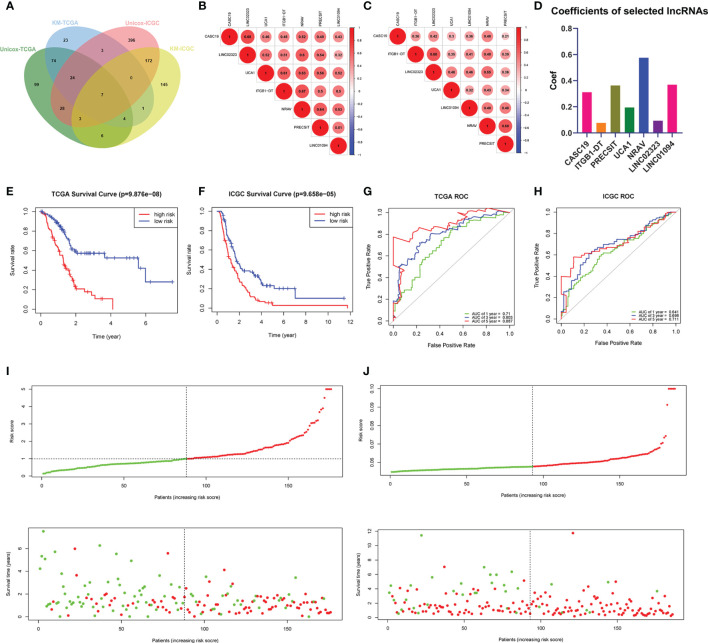
**(A)** Venn’s diagram screened the critical prognostic lncRNA-signatures in GTEx-TCGA and ICGC databases. **(B, C)** The correlation heatmap of the GTEx-TCGA database **(B)** and ICGC database **(C)**. **(D)** Used the LASSO regression to calculate the coefficients. **(E, F)** K-M curves showed that the high-risk subgroup had worse overall survival than the low-risk subgroup in TCGA **(E)** and ICGC **(F)** databases. **(G, H)** ROC curves of lncRNA-signatures for predicting the 1/3/5-year survival in the TCGA **(G)** and ICGC **(H)** databases. **(I, J)** The risk scores and survival status of PDAC patients were distributed in the TCGA **(I)** and ICGC **(J)** databases.

Firstly, using the downloaded profile from the “Ensembl” website, we identified 4441 lncRNAs in the GTEx-TCGA database and 14181 lncRNAs in the ICGC-CA database based on recognizing the Ensemble IDs of the genes for the following analysis. In addition, we extracted the expression matrixes of 26 m6A regulatory genes from the GTEx-TCGA and the ICGC-CA databases. A lncRNAs whose expression value was correlated with one of the 26 m6A regulatory genes, with | Pearson R| > 0.6 and p < 0.001 as the criterion, was defined as an m6A-related lncRNA. We obtained 762 lncRNAs significantly correlated with m6A regulatory genes in the GTEx-TCGA database, while 1370 lncRNAs in the ICGC database. Combined with the survival information, univariate Cox regression and Log-rank test were executed to screen seven m6A-related prognostic lncRNAs in both databases (**Figure 1A**).

### Establish the m6a-Related LncRNAs Prognostic Model in the TCGA Database

All m6A-related lncRNAs showed positive co-expression, which presented an extensive synergy effect (**Figures 1B, C**). The lncRNA CASC19 and LINC02323 possessed the most significant correlation coefficient (0.68) in the GTEx-TCGA database, while the lncRNA ITGB1-DT and LINC02323, NRAV and PRECSIT had the largest correlation coefficient (0.68) in the ICGC database. In order to build the m6A-related lncRNAs prognostic model for forecasting the overall survival of PDAC patients, at the same time, avoid the expression overfitting possibility brought by high correlation, we performed a LASSO regression analysis based on the seven m6A-related prognostic lncRNAs in the TCGA cohort. Moreover, it generated the prognostic model containing seven m6A-related lncRNAs and each lncRNA coefficient (**Figure 1D** and **Supplementary Figures 1A, B**). For each patient in the TCGA database, a risk score was calculated based on the coefficient for each lncRNAs. Patients in the TCGA training cohort were divided into low-risk and high-risk subgroups based on the median value of risk scores. K-M curves demonstrated that PDAC patients with higher risk scores had worse outcomes (**Figure 1E**). The ROC curves illustrated that the lncRNAs prognostic model has an excellent predictive ability to predict overall survival in the TCGA training cohort (1-year AUC = 0.710, 3-year AUC = 0.803, 5-year AUC = 0.887; **Figure 1G**). Risk score and survival status distributions are plotted in **Figure 1I**.

### Validation of the LncRNAs Prognostic Model in the ICGC Database

To validate the lncRNAs prognostic model’s predictive ability based on the TCGA training set, we calculated risk scores for patients in the ICGC cohort using the same equation. PDAC patients in the ICGC database were assigned to low-risk and high-risk subgroups according to the median risk score. The results were consistent with the TCGA database findings: PDAC patients with higher risk scores had lower overall survival rates and a shorter overall survival time in the ICGC dataset (**Figure 1F**). The ROC curves also demonstrated that m6A-related lncRNAs prognostic model had a robust prognostic value for PDAC patients in the ICGC database (1-year AUC = 0.641, 3-year AUC = 0.698, 5-year AUC = 0.711; **Figure 1H**). Risk score and survival status distributions are shown in **Figure 1J**, and it showed that patients with higher risk scores had shorter overall survival time and more death status. These results showed that the lncRNAs prognostic model was a robust and stable overall survival predictive tool.

### Prognostic Analysis of the Seven m6A-Related LncRNAs

**Figure 2:**
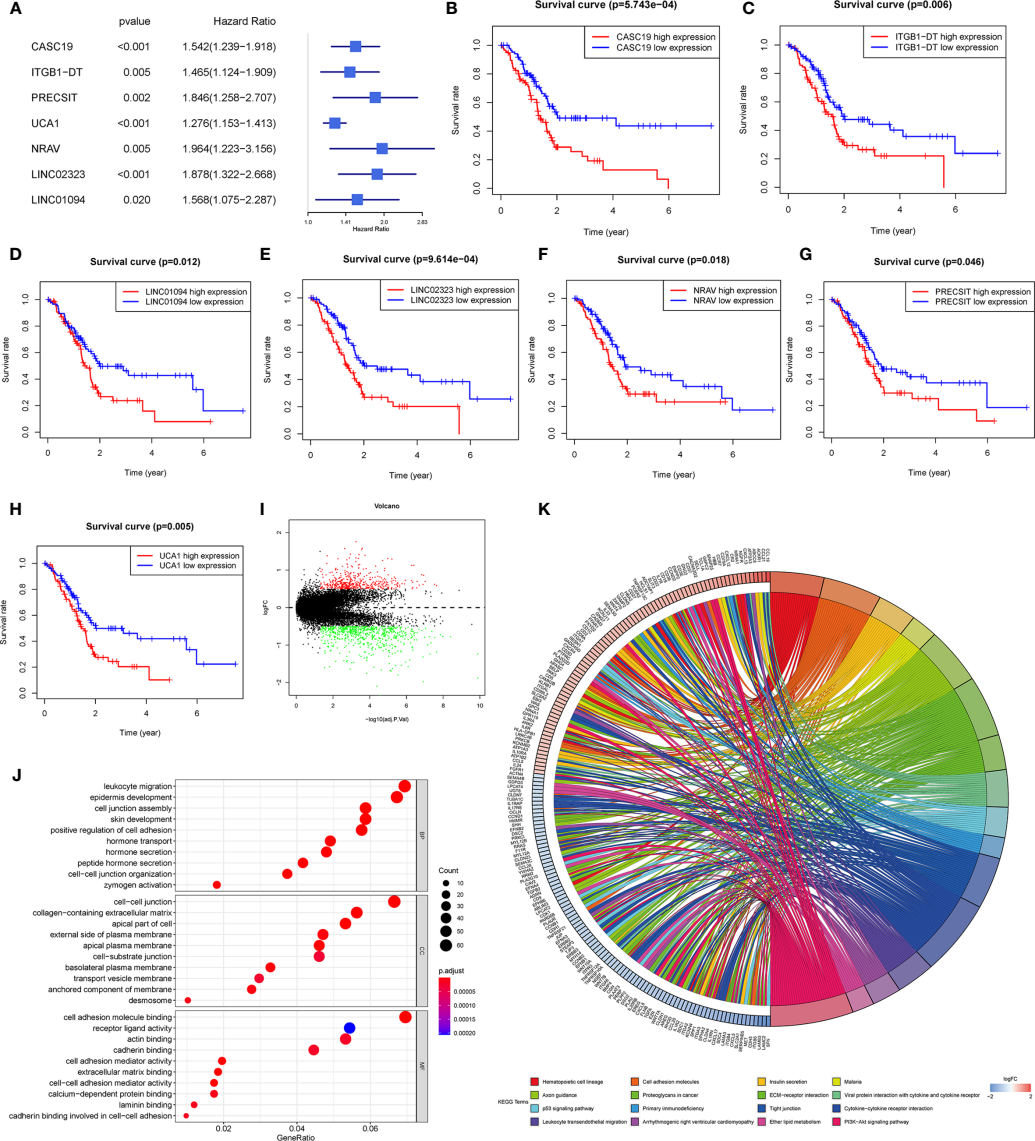
**(A)** Forest plot of the prognostic ability of the seven lncRNAs signatures. **(B–H)** K-M curves showed that patients with high expression levels of the seven lncRNAs signatures had worsened overall survival. **(I)** Differential genes were extracted from patients with different risk subgroups. **(J, K)** GO **(J)** and KEGG **(K)** analyses were performed for different risk subgroups.

Univariate Cox regression analysis was employed to evaluate seven m6A-related lncRNAs in the prognostic model and their prognostic roles. The forest plot shows that all of them are risk factors with Hazard Ratio (HR) >1 in PDAC patients (**Figure 2A**). The K-M survival curves confirmed that higher expression of CASC19, ITGB1-DT, LINC01094, LINC02323, NRAV, PRECSIT, and UCA1 were associated with worse overall survival in the TCGA database (**Figures 2B–H**).

### Pathway and Process Enrichment Analysis of Different Risk Subgroups

For investigating the potential biological process and pathway involved in the molecular heterogeneity between the low-risk and high-risk subgroups, we identified 1107 differential expression genes (DEGs) between the low-risk and high-risk subgroups in the TCGA cohort with the filter criteria |log2 (fold change)| > 0.5 and p < 0.05 (**Figure 2I**). These DEGs were primarily enriched in these GO terms: the biological process (BP) included the leukocyte migration, epidermis development, cell junction assembly, skin development, positive regulation of cell adhesion, etc.; the cell component (CC) contains cell-cell junction, collagen-containing extracellular matrix, apical part of the cell, external side of the plasma membrane, apical plasma membrane, etc.; the molecular function (MF) included cell adhesion molecule binding, receptor-ligand activity, actin binding, cadherin binding, cell adhesion mediator activity, etc. (**Figure 2J**). The KEGG analyses revealed that 16 malignant characteristics were enriched in the high-risk subgroup, such as hematopoietic cell lineage, cell adhesion molecules, insulin secretion, malaria, axon guidance, etc. (**Figure 2K**). These results may disclose some perspectives into the cellular biological effects related to the m6A-related lncRNAs prognostic model.

### Stratification Analysis of the m6A-Related LncRNAs Prognostic Model

**Figure 3:**
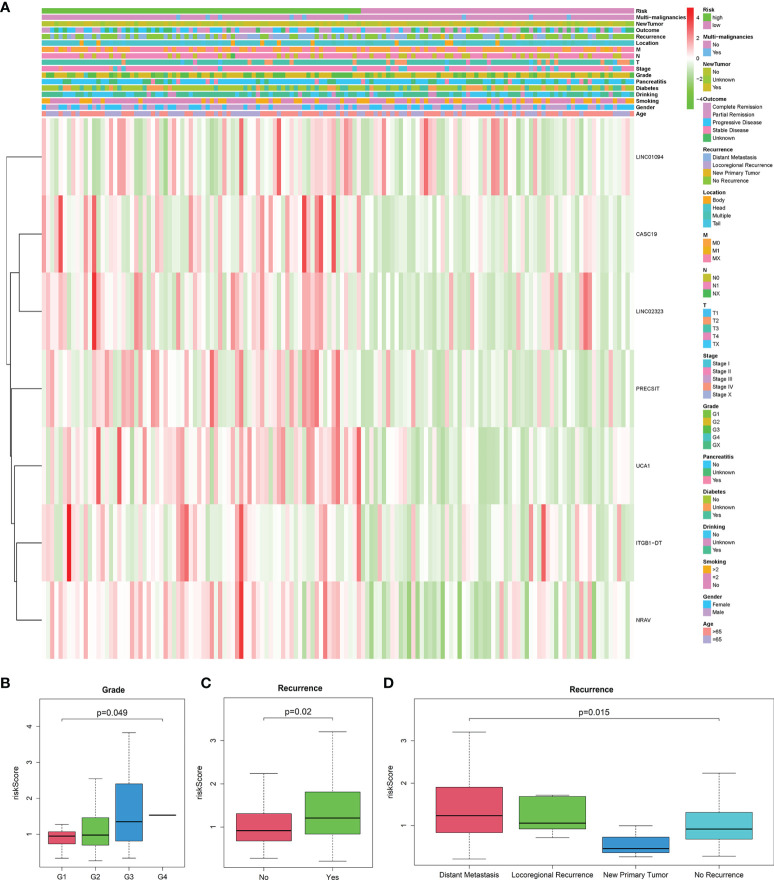
**(A)** Heatmap of the connections between the expression levels of the seven lncRNAs signatures and clinicopathological features in the TCGA database (n=141). **(B–D)** Patients with different clinicopathological features, including pathology grade (G1 = 18, G2 = 82, G3 = 39, G4 = 1, GX=1) and recurrence event (Distant Metastasis=51, Locoregional Recurrence=16, New Primary Tumor=3, No Recurrence=71), had different levels of risk scores, calculated based on the seven lncRNAs signatures.

**Figure 4:**
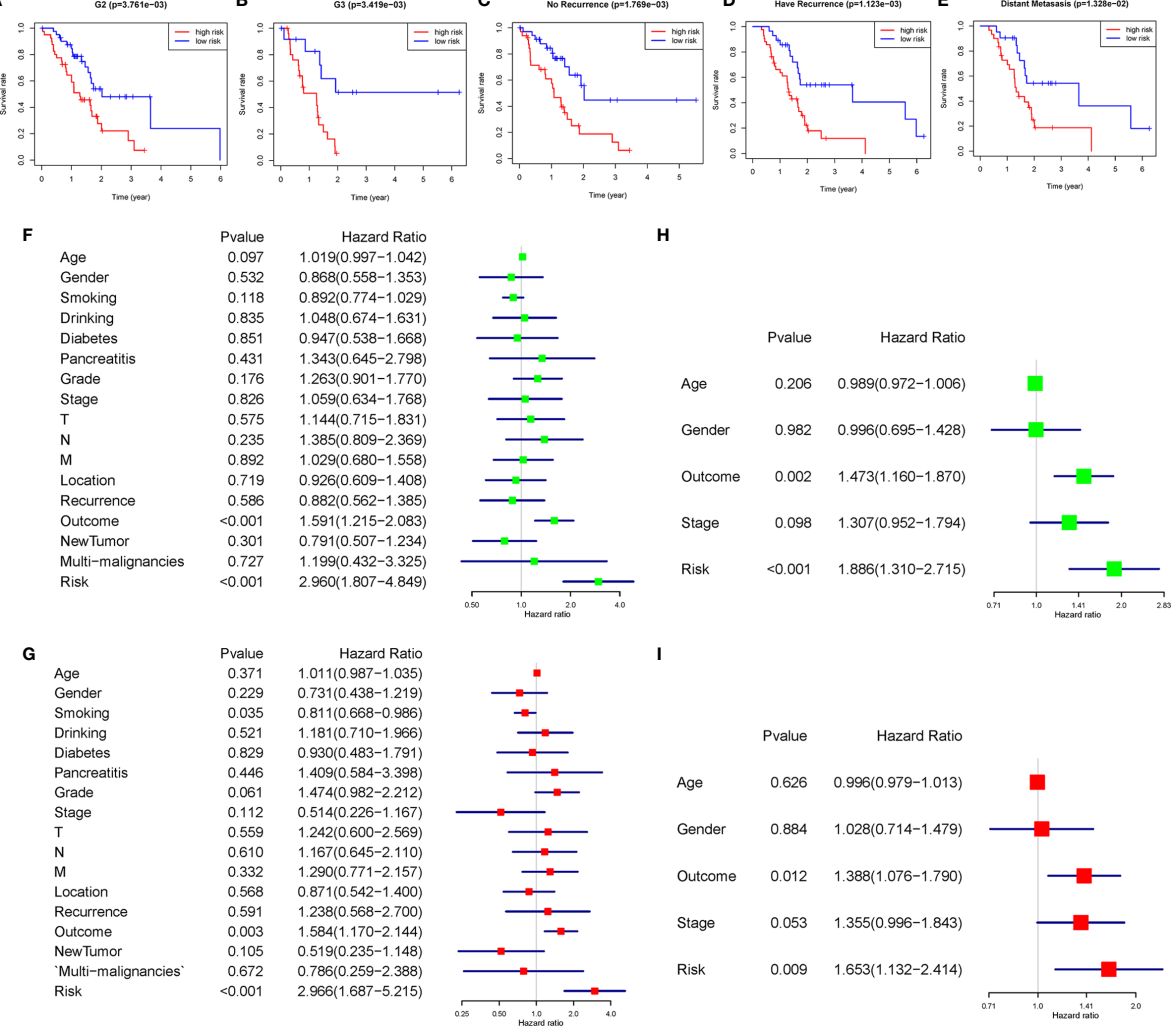
**(A–E)** The seven lncRNA-signatures retained their prognostic value in multiple subgroups of PDAC patients, including patients with pathology grade two, pathology grade three, no recurrence event, have recurrence event, and distant metastasis. **(F–I)** Univariate and multivariate analyses revealed that risk stratification was an independent prognostic predictor in the TCGA **(F, G)** and ICGC **(H, I)** databases.

The heatmap exhibited LINC01094, CASC19, LINC02323, PRECSIT, UCA1, ITGB1-DT, and NRAV were enriched in the high-risk subgroup and showed the association between each m6A-related lncRNAs expression and the clinicopathological features of PDAC patients (**Figure 3A**). We attempted to identify whether clinicopathological characteristics were connected with the risk score (**Figures 3B–D** and **Supplementary Figures 2A–N**). The results revealed that the PDAC patients with a higher risk score, the tumor issue have a worse pathological differentiation and showed a strictly increasing relationship (P<0.05). Besides, we found that the PDAC patients with higher risk scores were also more likely to have tumor recurrence after treatment (P<0.05). The patients with distant metastasis had the highest risk score, followed by the locoregional recurrence. The new primary tumor had the lowest risk score with only three samples. To better assess the m6A-related lncRNAs prognostic model’s prognostic capacity, we carried out a stratification analysis to verify whether it can forecast overall survival in various subgroups. We performed K-M curves for subgroups with more than five people’s risk stratification according to the clinicopathological characteristics. In contrast with lower risk score patients, higher risk PDAC patients had worse overall survival in both the pathological G2 and G3 grades (**Figures 4A, B**), while there was no significant difference in the G1 stratification (**Supplementary Figure 3A**). Likewise, we confirmed that the m6A-related lncRNAs prognostic model retained its capacity to predict overall survival for whether PDAC patients have recurrence events or not (P<0.05, **Figure 4C, D**). Moreover, the further detailed stratification was performed, we detected that the PDAC patients with distant metastasis had a shorter survival time(P<0.05, **Figure 4E**). On the contrary, the locoregional recurrence patients had no significant difference (**Supplementary Figure 3B**).

### LncRNAs Prognostic Model Was an Independent Prognostic Factor for PDAC Patients

We used univariate and multivariate Cox analyses to assess whether the m6A-related lncRNAs prognostic model was an independent prognostic factor for patients with PDAC. Based on the data of PDAC patients in the TCGA database, univariate Cox analysis indicated that lncRNAs prognostic model was remarkably associated with overall survival [the hazard ratio (HR): 2.960, 95% confidence interval (CI): 1.807-4.849, p < 0.001; **Figure 4F**] and multivariate Cox analysis further showed that lncRNAs prognostic model was an independent predictor of overall survival, with the HR(95%CI) was 2.966(1.687-5.215) (p < 0.001, **Figure 4G**). The same results were verified in the ICGC database with less clinicopathological characteristics abundance (**Figures 4H, I**). These results indicated that the m6A-related lncRNAs prognostic model might avail clinical prognosis evaluation as an independent prognostic indicator.

### Construction of the ceRNA Network and Functional Enrichment Analysis

**Figure 5:**
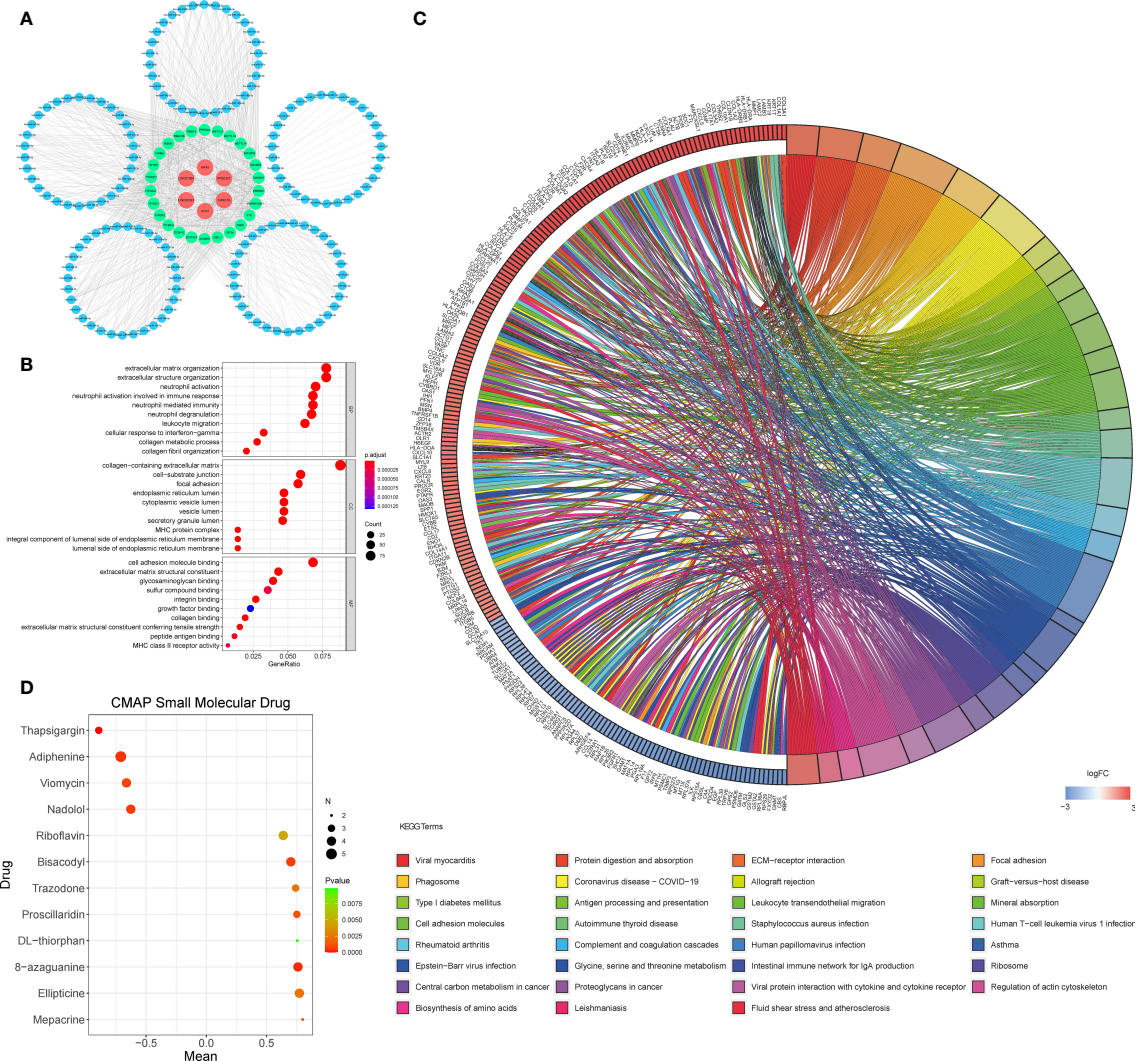
**(A)** The ceRNA network of the six m6A-related lncRNAs (red) and their target miRNAs (blue) and m6A-related methylation regulative mRNAs (green). **(B, C)** GO **(B)** and KEGG **(C)** analyses were performed for differential target mRNA expression. **(D)** CMAP was used to explore the potential drug to cure PDAC according to the expression level of targeted mRNA in the ceRNA network in the GTEx-TCGA database (P< 0.01,∣mean∣> 0.6).

To further understand how the critical lncRNAs act on N6-methyladenosine regulators by sponging miRNAs in PDAC patients, we constructed a ceRNA network to explore the mechanism of m6A-related lncRNAs. Six lncRNAs were extracted from the Starbase and miRcode databases, and 162 pairs of interactions between the 6 lncRNAs and 153 miRNAs were identified. Then we excavated three databases (Starbase, miRDB, and miRTarBase) to search target N6-methyladenosine regulators based on the 153 miRNAs and a total of 890 pairs of interactions between the 153 miRNAs and 26 m6A regulators were identified in all three databases. Finally, 6 lncRNAs, 153 miRNAs, and 26 m6A regulators were included in the ceRNA network (**Figure 5A**). Furthermore, there are 17288 mRNAs we extract from the three databases the 153 miRNAs target, and we affirmed 1145 DEGs from the GTEx-TCGA database with the filter criteria |log2 (fold change)| > 2 and p < 0.01. These DEGs were wielded to implement functional enrichment analysis, and we found that these genes were enriched in the BP included extracellular matrix organization, extracellular structure organization, neutrophil activation, etc.; the CC contains collagen-containing extracellular matrix, cell-substrate junction, focal adhesion, etc.; the MF included cell adhesion molecule binding, extracellular matrix structural constituent, glycosaminoglycan binding, etc. (**Figure 5B**). KEGG analysis showed that 31 signaling pathways were enriched in pancreatic cancer, some of which had tumor characteristics, including protein digestion and absorption, ECM-receptor interaction, focal adhesion, etc (**Figure 5C**). These data may provide medical workers clues for finding the potential pathways of these m6A-related lncRNAs in PDAC.

### Exploration of Small Molecule Drugs Related to Pancreatic Cancer

We put the 1145 DEGs mentioned above into the CMAP database for analysis. We set P< 0.01,∣mean∣> 0.6 as the filter criteria, and extracted twelve small molecule drugs related to PDAC. The Thapsigargin, Adiphenine, Viomycin, and Nadolol negative control the m6A-related lncRNAs targeted mRNA expression. While the Mepacrine, Ellipticine, 8-azaguanine, DL-thiorphan, Proscillaridin, Trazodone, Bisacodyl, and Riboflavin positive regulated the targeted mRNA expression level (**Figure 5D**).

### The Differential Expression Level of Pivotal m6A-Related LncRNAs

**Figure 6:**
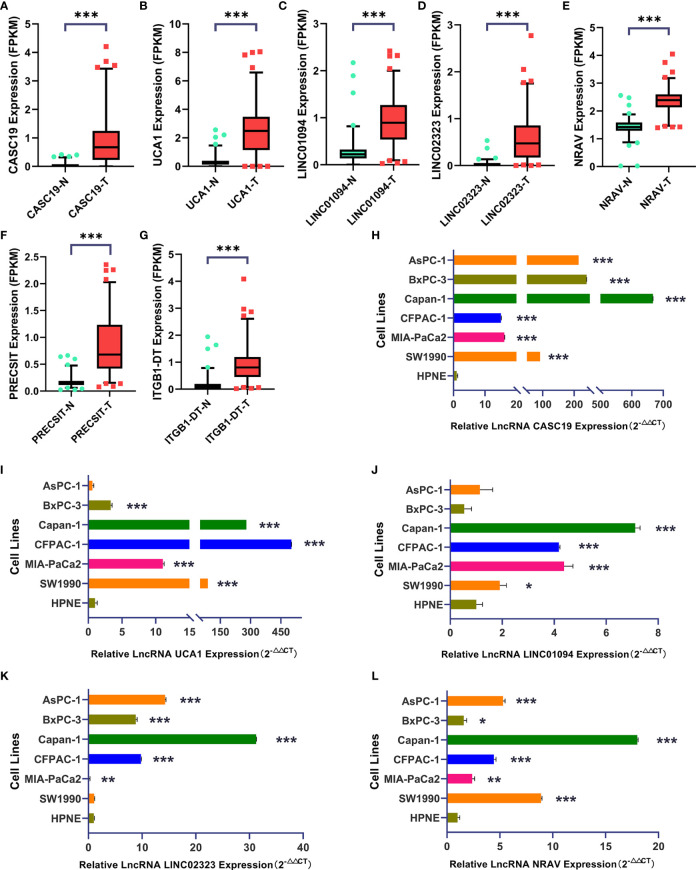
**(A–G)** Seven lncRNAs signatures were quantified in tumor and normal tissues using the GTEx-TCGA database (T=178, N=171). **(H–L)** LncRNA CASC19/UCA1/LINC01094/LINC02323/NRAV expression in six PDAC cell lines and one pancreatic duct epithelial cell line (Triplicate). The symbol * means P < 0.05, ** means P < 0.01, *** means P < 0.001.

**Figure 7:**
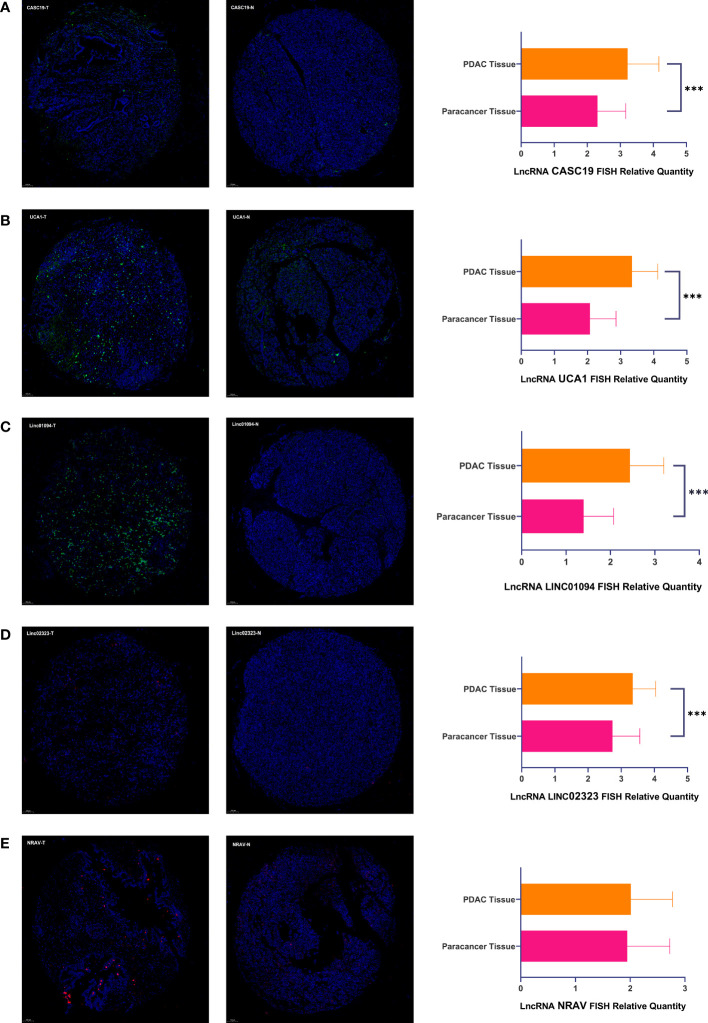
**(A–E)** There were representative microphotographs and relative quantity of PDAC tissues and paracancer normal tissues using tissue microarray-based on FISH about five pivotal lncRNAs (PDAC Tissue=90, Paracancer Tissue=90). The symbol *** means P < 0.001.

We analyzed the expression of each m6A-related lncRNAs in PDAC patients compared to normal pancreas tissues in the GTEx-TCGA database using the R package “limma.” Based on the Bayesian algorithm, we generated boxplots for seven critical lncRNAs and observed a statistically significant increased expression in tumor samples for all m6A-related lncRNAs (p < 0.001, **Figures 6A–G**). We used six PDAC cell lines and one normal pancreatic duct epithelium cell line to explore seven lncRNAs expression. Significant differences existed among these cell lines in LncRNAs CASC19/UCA1/LINC01094/LINC02323/NRAV based on the student’s t-test, which were following the bioinformatic analysis (**Figures 6H–L**). In contrast, the results about LncRNA PRECSIT and ITGB1-DT were not consistent with the above (**Supplementary Figures 3C, D**). FISH staining was performed on tissue microarray to confirm whether critical lncRNAs also differ in human tissues. The results showed that the LncRNA CASC19/UCA1/LINC01094/LINC02323 relative quantity of PDAC was higher than paracancer normal tissues based on the Mann-Whitney test(p < 0.001, **Figures 7A–D**). The LncRNA NRAV FISH relative quantity was slightly higher in PDAC tissues than in paracancer normal tissues, but there was no statistical difference (**Figure 7E**).

## Discussion

A total of 440 PDAC tumor samples and 171 normal tissues from the GTEx-TCGA and ICGC cohorts were included in our study to exploit the prognostic significance of m6A-related lncRNAs. Seven pivotal lncRNAs were confirmed to have prognostic value in both the TCGA and ICGC databases, and were used to establish an m6A-related lncRNAs prognostic model for predicting the overall survival of PDAC patients. Based on each cohort’s median risk score, PDAC patients were divided into the low-risk and high-risk subgroups, and the high-risk group had worse clinical outcomes and enrichment of neoplasm characteristics and specific malignant-related pathways. The higher patients’ risk score, the worse the pathological grade and the more recurrent events. Multivariate Cox regression analysis showed that the m6A-related lncRNAs prognostic model and initial treatment outcome were the independent risk factors for overall survival. A ceRNA network consisted of 6 m6A-related lncRNAs, 153 miRNAs, and 26 m6A regulators to view this lncRNAs prognostic model’s potential functions. Simultaneously, we analyzed the targeted mRNA’s enrichment analysis to discover its potential biological function and signal pathway based on the m6A-related lncRNAs prognostic model. Moreover, we found that Thapsigargin, Adiphenine, Viomycin, and Nadolol negative control the m6A-related lncRNAs targeted mRNA expression. Besides, the Mepacrine, Ellipticine, 8-azaguanine, DL-thiorphan, Proscillaridin, Trazodone, Bisacodyl, and Riboflavin positive regulated the targeted mRNA expression level. These tiny molecular drugs may cure pancreatic cancer by intervening in the m6A-related ceRNA network expression. Meanwhile, we found that seven lncRNAs were highly expressed in tumor samples in the GTEx-TCGA database. In order to confirm the robustness of the prognostic model and strive for application to clinical translational, we performed experimental validation of critical lncRNAs. The cell experiments confirmed that the five m6A-related lncRNAs in PDAC samples was overexpressed than the normal pancreas tissues and seven cell lines, and the FISH staining further demonstrated that the lncRNAs CASC19/UCA1/LINC01094/LINC02323 highly expressed in the tissue microarray, which is good for clinical workers to screen and diagnose PDAC patients.

Multiple projects have suggested that m6A modification might function as a regulator in oncogenicity, but it is still unclear how it acts in a lncRNA-dependent pattern during PDAC progression. To date, m6A regulators can maintain the malignancy of PDAC by modifying specific lncRNAs has only been mentioned in a few articles. He et al. have found ALKBH5 inhibits pancreatic cancer motility by demethylating lncRNA KCNK15-AS1 (13). The research may be the earliest experimental exploration of how lncRNA affects pancreatic cancer through m6A modification. Shortly after that, Hu et al. demonstrated that lncRNA DANCR targets IGF2BP2 through m6A modification, and IGF2BP2 and DANCR work together to promote cancer stemness-like properties and pancreatic cancer pathogenesis (12). Meng et al. revealed N6-Methyladenosine was highly enriched within LINC00857 and enhanced its RNA stability. Meanwhile, LINC00857 modulates E2F3 expression by binding to miR-150-5p, ultimately promoting tumorigenesis in pancreatic cancer (23). Studies had disclosed that m6A modification of lncRNAs could influence pancreatic cancer tumorigenesis, and lncRNAs might serve as competing endogenous RNAs, targeting m6A regulators and influencing aggressive tumor progression. Based on the above considerations, we believe that lncRNAs participated in m6A modification, and we ought to pay more attention to the interactions and functions of lncRNAs and m6A modifications to identify prognostic markers and therapeutic targets of pancreatic cancer.

We identified seven m6A-related prognostic lncRNAs from three databases and 611 samples. The lncRNA CASC19 developed pancreatic cancer with CASC19/miR-148b/E2F7 axis (24). Multiple studies have indicated that UCA1 acts as a ceRNA in developing and progressing pancreatic cancer in multiple axes (25–27). LINC02323 sponged miR-1343-3p to upregulate the TGFBR1 expression and promote the epithelial-mesenchymal transition and metastasis in lung adenocarcinoma (28). PRECSIT promotes the progression of cutaneous squamous cell carcinoma *via* STAT3 signaling (29). LINC01094 facilitates clear cell renal cell carcinoma radioresistance by targeting the miR-577/CHEK2/FOXM1 axis (30). NRAV has a certain suggestive effect in predicting the prognosis of hepatocellular carcinoma (31). ITGB1-DT could form a positive feedback loop with ITGB1/Wnt/β-Catenin/MYC to facilitate lung adenocarcinoma progression (32). Seven lncRNAs were reported to be associated with oncogenesis, but there have been few reports regarding pancreatic cancer and no reports on how the lncRNAs interact with the m6A regulator so far. Our study identified the seven pivotal m6A-related prognostic lncRNAs, thereby providing insights into their potential roles in PDAC tumorigenesis and progression.

In our research, pathological grades and recurrent events were associated with higher risk scores, indicating a poor prognosis. Histopathological grading of PDAC is an important prognostic factor that has become the consensus of surgeons (33). Besides, metastasis causes more significant than 90% of cancer death, although recent therapeutic advances in cancer treatment (34). Fortunately, we have access to some small molecule drugs with therapeutic potential through the CMAP database. We found that the positively regulated drug mepacrine has been initially used as an antimalarial. Moreover, a growing body of reports suggested that mepacrine was associated with gynecologic and breast cancer treatment (35). Recent research shows that treatment of pancreatic cancer by carefully packaged nanoparticles enhances the activity of Mepacrine and minimizes their associated hepatotoxicity (36, 37). Ellipticine and its derivatives were isolated from the wood of Ochrosia elliptica, which showed clinical responses in AML, thyroid cancer, renal cancer, and bone metastases from advanced breast cancer and soft−tissue sarcoma in clinical trials (38). The novel immunomodulatory drug 8-azaguanine significantly increased the cytotoxicity of NK cells and developed as an antineoplastic agent (39). On the other hand, the negative control group molecule thapsigargin could induce apoptosis by inhibiting the sarcoplasmic/endoplasmic reticulum Ca^2+^ ATPase pump, which can treat as a novel antineoplastic agent. However, the high toxicity of this compound to normal cells is a significant impediment (40). Adiphenine is an anticholinergic drug, but there is no reported use in the treatment of cancer currently. Viomycin is a tuberactinomycin antibiotic that could inhibit bacterial protein synthesis by blocking elongation factor G catalyzed translocation of mRNA on the ribosome. It is essential for treating multi-drug-resistant tuberculosis, but there is no literature on cancer treatment at present (41). Ultimately, we demonstrated that four pivotal lncRNAs were overexpressed coherently in patients, cell lines, and tissue microarray, which means that these four markers can be specifically screened for pancreatic cancer in clinical applications.

Although we built a robust and reliable model, there were several limitations in our study. First, the K-M curves of patients with G1 and G3 pathologic grades intersect between half and one year because most patients received surgical treatment. In the short term, surgical treatment patients are at risk of complications that lead to death after surgery, resulting in a bias. In the long term, low-risk patients had longer survival times across all pathological stratifications. Another hand, Pancreatic cancer is relatively rare compared to other cancers, and the lack of data to analyze may also lead to bias. Moreover, the lncRNAs’ role and their interactions with m6A regulators should be confirmed through experiments, which is the next step for our team to explore. Lastly, the staining method of qRT-PCR has some limitations in clinical application because of its low detection sensitivity, which may be why PRECSIT and ITGB1-DT cannot be consistent with the results from the GTEx-TCGA database. Furthermore, the abundance of lncRNA in peripheral blood is lower than that in cells, so it is necessary to improve the sensitivity of detection methods for better application in clinical detection. What’s more, the FISH staining showed that the lncRNA NRAV had no significant difference between PDAC and paracancer tissues, the poor quality of tissue microarray and insufficient sample quantity may be the main reasons.

There are few studies on how lncRNAs affect the progression of pancreatic cancer through the m6A modification. First of all, our project has filled the gap in predicting clinical prognosis based on m6A-related lncRNAs. We established a robust and effective m6A-related lncRNAs prognostic model for pancreatic cancer in multiple databases. Secondly, we analyzed the relationship between risk score and abundant clinicopathological characteristics based on the prognostic model. Moreover, we analyzed the possible biological mechanism and signaling pathway of the pivotal m6A-related lncRNAs and found its ceRNA network between lncRNAs and m6A-regulators through miRNAs. Thus, we could find out small molecule drugs that may treat pancreatic cancer by targeting the ceRNA network, providing more attractive clues for researchers studying pancreatic cancer. In the end, we used cell experiments and FISH staining to confirm that the expression of key lncRNA in the prognostic model was significantly higher in PDAC than the normal sample, thus providing compelling evidence for clinical translation, which is also ahead of similar studies in the field. Considering that our team will apply this result to clinical trials later, we will use the probe method to accurately detect critical m6a-related lncRNA in blood for clinical application of pancreatic cancer in the future.

## Conclusion

We developed a robust m6A-related lncRNA prognostic model for clinical workers to predict PDAC overall survival. Moreover, the stratification analysis demonstrated that the higher risk score was associated with the worse pathological grade and the more recurrent events. Furthermore, the enrichment analysis indicated that malignancy-associated biological function and signaling pathways were enriched in the high-risk subgroup and m6A-related ceRNA network. Besides, the small molecule drugs that may affect the progression of PDAC were identified. Ultimately, we confirmed that the LncRNA CASC19/UCA1/LINC01094/LINC02323 were overexpressed in the GTEx-TCGA database, pancreatic cell lines, and tissue microarray. In conclusion, we provided a comprehensive aerial view between m6A-related lncRNAs and pancreatic cancer’s clinicopathological characteristics and performed experiments to verify the robustness of the prognostic model.

## Data Availability Statement

Publicly available datasets were analyzed in this study. This data can be found here: the University of California, Santa Cruz (UCSC) website (https://xenabrowser.net/datapages/), The Cancer Genome Atlas (https://portal.gdc.cancer.gov/), and International Cancer Genome Consortium (https://icgc.org).

## Ethics Statement

The studies involving human participants were reviewed and approved by The Ethics Committee of Peking Union Medical College Hospital. The patients/participants provided their written informed consent to participate in this study.

## Author Contributions

BWH conceived the study. BWH, JZL, and JL wrote the manuscript. WYG, LZ, FT, and YZW applied to guide suggestions. MJL, DL, CYX, and ZYX analyzed the data. CXL, YW, and HBM prepared the dataset. JCG obtained financial support. All authors contributed to the article and approved the submitted version.

## Funding

This study was supported by the National Natural Science Foundation of China (Grant No. 81972324), the Chinese Academy of Medical Sciences Innovation Fund for Medical Sciences (Grant No. 2016-I2M-3-019), and the non-profit Central Research Institute Fund of Chinese Academy of Medical Sciences (Grant No. 2018PT32014). The funder bodies were not involved in the study design, collection, analysis, interpretation of data, the writing of this article or the decision to submit it for publication.

## Conflict of Interest

The authors declare that the research was conducted in the absence of any commercial or financial relationships that could be construed as a potential conflict of interest.

## Publisher’s Note

All claims expressed in this article are solely those of the authors and do not necessarily represent those of their affiliated organizations, or those of the publisher, the editors and the reviewers. Any product that may be evaluated in this article, or claim that may be made by its manufacturer, is not guaranteed or endorsed by the publisher.

## Supplementary Material

Supplementary Figure 1
**(A, B)** Used the LASSO regression to calculate the minimum value of lambda.

Supplementary Figure 2
**(A–N)** Patients with different clinicopathological features had different levels of risk scores, but there was no statistically significant difference.

Supplementary Figure 3
**(A)** The K-M curve of the G1 subgroup. **(B)** The K-M curve of the locoregional recurrence subgroup. **(C, D)** LncRNA PRECSIT/ITGB1-DT expression in six PDAC cell lines and one pancreatic duct epithelial cell line.
