# Supplementary figures and images for: Aerial View of the Association Between m6A-Related LncRNAs and Clinicopathological Characteristics of Pancreatic Cancer
Source: Front Oncol. 2022 Jan 3;11:812785. doi: 10.3389/fonc.2021.812785 (PMC8762256; doi:10.3389/fonc.2021.812785)

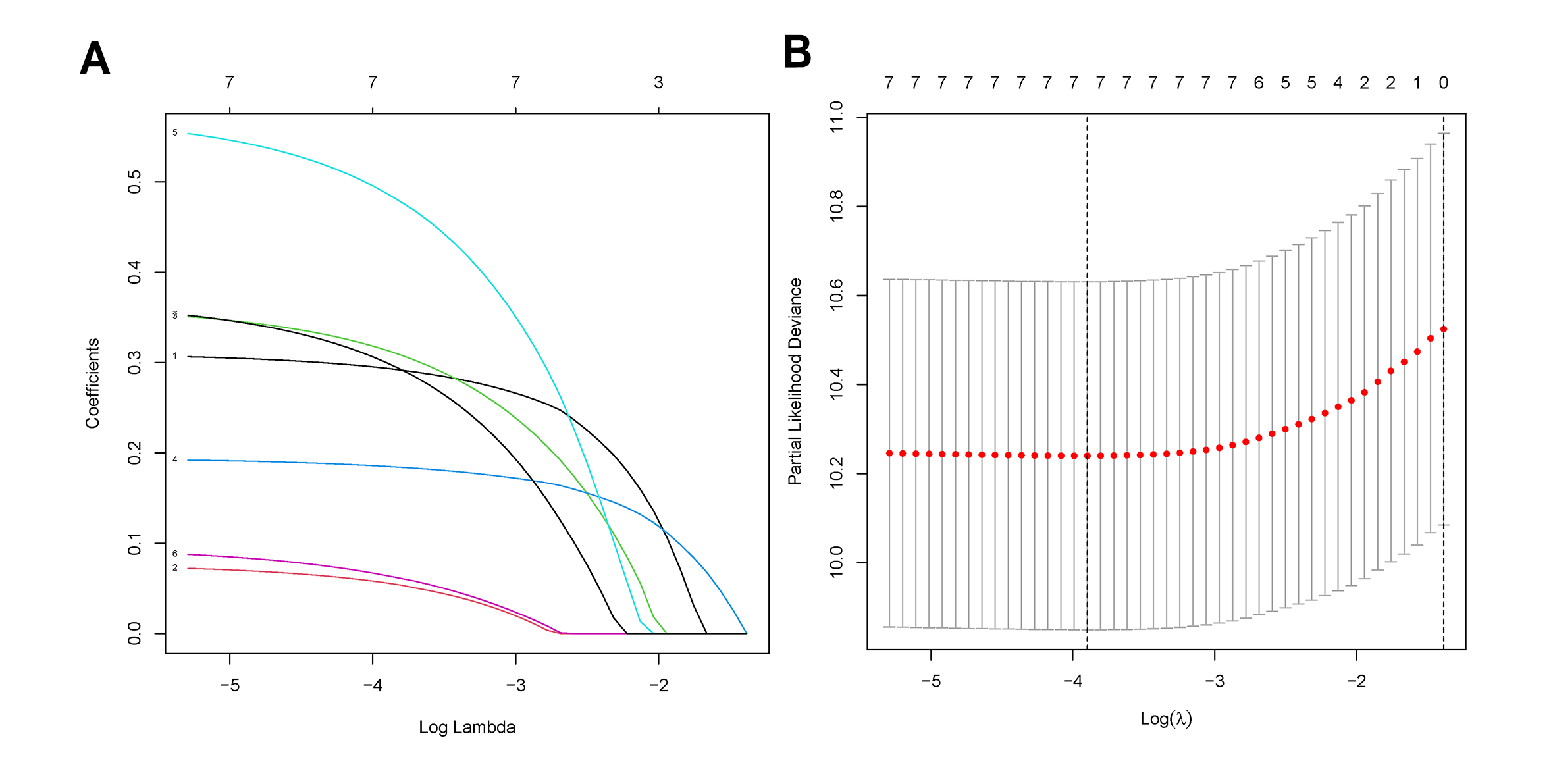

Supplement: Supplementary Figure 1 — (A, B) Used the LASSO regression to calculate the minimum value of lambda. [file Image_1.tif]

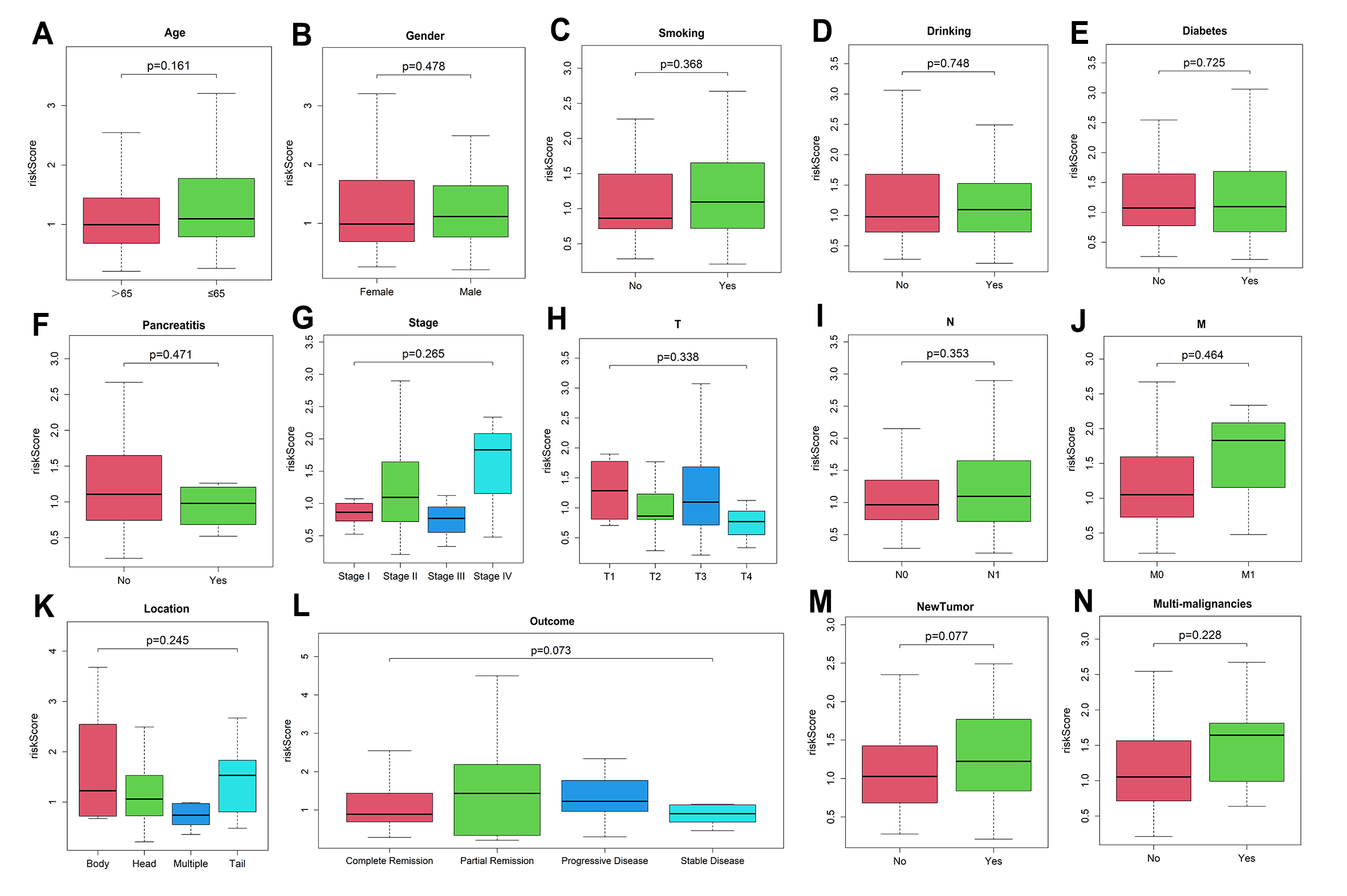

Supplement: Supplementary Figure 2 — (A–N) Patients with different clinicopathological features had different levels of risk scores, but there was no statistically significant difference. [file Image_2.tif]

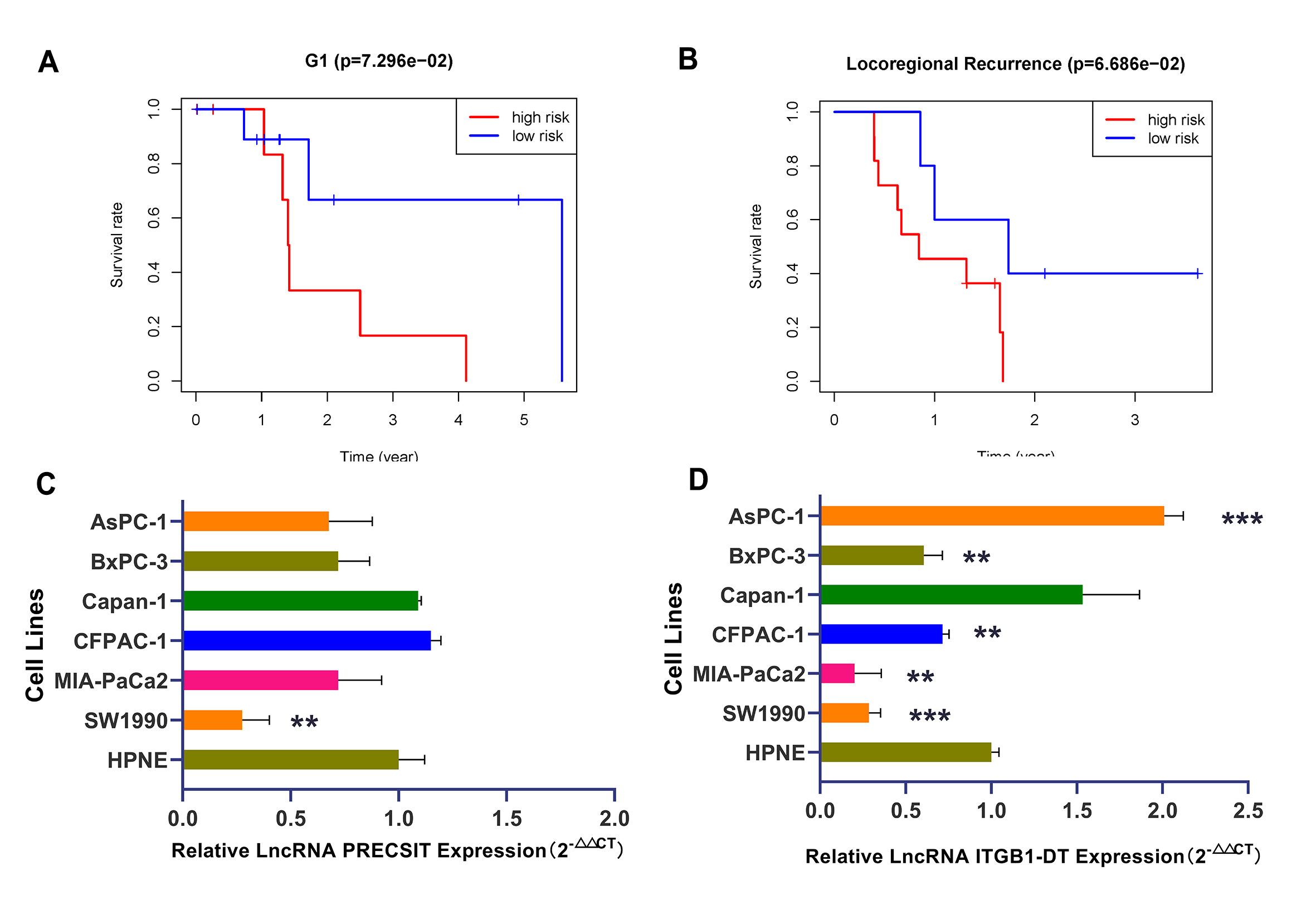

Supplement: Supplementary Figure 3 — (A) The K-M curve of the G1 subgroup. (B) The K-M curve of the locoregional recurrence subgroup. (C, D) LncRNA PRECSIT/ITGB1-DT expression in six PDAC cell lines and one pancreatic duct epithelial cell line. [file Image_3.tif]
