# Supplementary material for: Aerial View of the Association Between m6A-Related LncRNAs and Clinicopathological Characteristics of Pancreatic Cancer
Source: Front Oncol. 2022 Jan 3;11:812785. doi: 10.3389/fonc.2021.812785 (PMC8762256; doi:10.3389/fonc.2021.812785)
Supplement: Supplementary file 4 [file Table_1.docx]

Supplement Table 1. The primers sequences were listed below.

| Gene | Forward Primer (5' - 3') | Forward Primer (5' - 3') |
| --- | --- | --- |
| CASC19 | CCTGGGTTAGAACCCTGCTG | TGGACAGCACCTTGAATGCT |
| LINC02323 | TTTGTCCAGGACGTTCCCTC | CCGGTCCCGGATTTAAGCAG |
| UCA1 | TCATCGGCTTAGCAACAGGG | TTTTGAGCTTGGAACTGCCC |
| ITGB1-DT | CCTGAGTCCCGAGGCAAATC | CAGTCTCACCACCCTTCGTG |
| NRAV | ATCACCTTTCAGCTCCGGTC | CTGGGGCAGTAGTGAGGAAC |
| PRECSIT | AAGAACAAGCCCCAAGCTGT | TGGGGTGACAGACGCATTAC |
| LINC01094 | AGCCTCGGCTGTGTTTGTAT | AGGTTGACACATCTCGCCTG |
| GAPDH | TCGGAGTCAACGGATTTGGT | TTCCCGTTCTCAGCCTTGAC |
